# The Vomiting of Pregnancy

**Published:** 1912-02-10

**Authors:** 


### MATERNITY AND MOTHERING.

(Inquiries and Correspondence are invited.)

### The Vomiting of Pregnancy.

There are many inconveniences from which at different periods an expecting mother may be liable to suffer. Fortunately nearly all of these are uncommon, except two; those two are constipation and vomiting. The former may be troublesome at any stage of progress, and its treatment is essentially similar in principle to that of the same disorder at any other time. The latter is a symptom the causation of which is still extremely obscure; and it follows that the treatment is necessarily in large measure empirical.

The vomiting of pregnancy affects the majority of those who conceive; though there is a quite fairly large minority who escape it. In ordinary cases it commences in the second month, ceases in the fourth, and is most troublesome in the third. It is capricious in occurrence, making its appearance at no regular or calculable intervals. It is commonest in the early morning and after breakfast; but may also occur at other times, especially during or after a meal. It is often absent for several days, only to recommence as a result of dietary indiscretions, or for no obvious reason. Sometimes nausea and retching occur with little or no vomiting; but vomiting without nausea is distinctly uncommon. There is also the much rarer form known as pernicious or intractable vomiting, or as hyperemesis gravidarum. Fortunately this is exceedingly rare, for it is a most difficult condition to cope with, and taxes the resources of physician and patient alike. There is, however, no hard and fast line between a case that may be regarded as intractable and one that is simply a rather severe case of the common type. In the beginning there is nothing to distinguish one from the other: and that is one reason why early treatment of vomiting during pregnancy is always desirable. So many patients seem to regard the trouble as a necessary evil that very often they just put up with it without getting expert advice.

It has been stated that the cause of this vomiting is obscure; and this holds good in most cases. There are, however, abnormal conditions which may account for unusual degrees of this trouble; and that is another reason for seeking a professional opinion. Without entering into technical details, it may be said that in some cases excessive vomiting is a sign or warning of complications which call urgently for interference. What can be done to alleviate this common and distressing inconvenience?

### Treatment.

To begin with, the ordinary rules of health must be observed with more than ordinary care and vigilance. Fresh air, early and regular hours, sufficient exercise, strict moderation in eating and drinking, are absolutely essential. The regulation of the bowels is also of the greatest importance, and mild aperients must be taken to secure this regularity if other measures fail. If glasses have been advised at any time, and given up for cosmetic reasons, they should be resumed whenever any reading or sewing is being done. If the teeth are in any way defective a dentist should be consulted, even though there may be no toothache. In short, personal hygiene must receive the fullest attention.

As for drugs, the numbers that have been advised are legion. A very excellent prescription is one containing three or four minims of dilute hydrocyanic acid with ten to fifteen grains of sodium bicarbonate in an ounce of chloroform water. Sometimes an effervescing draught containing the same drugs seems to act even better. Another excellent preparation is the liquor bismuthi et ammon. citratis of the Pharmacopœia, to which may be added syrup of Virginian prunes; both of these should be given in liberal doses, if at all. Doses of one drop of tincture of iodine in an ounce or two of water are a favourite with some experienced practitioners. Cerium oxalate had a great vogue a few years ago: this, again, is of little use in small doses; five grains at least should be given at a time.

Another recently favoured remedy is calcium lactate, which seems to have been recommended for nearly every ill that women can have. The taste of this drug is so nauseous that it is best given in cachet. Morphine, or preparations containing it, are, as a rule, to be absolutely avoided. Champagne will sometimes quell vomiting which has resisted all treatment. Thyroid extract has been highly praised lately. The routine advised by a German author is as follows: Patient awakened at 5 A.M. for first dose, repeated at 9 A.M. after breakfast in bed, and again half an hour before the other meals and before retiring. The morning dose to be not less than five grains, the others smaller. Food to be taken only in small amounts at a time, meat interdicted.

Dr. Martin last year published * his experiences in a number of exceptionally severe cases. He found every single patient thus seriously affected to have bad teeth and chronic constipation. The patients were kept in bed, and stomach lavage with warm water was performed. Large quantities of mucus were thus removed from the stomach. Sips of cold water were then allowed, and a common enema was given at night. Powders containing one grain of hydrargyrum cum cretâ and three grains of sodium bicarbonate were given thrice a day, and Epsom salts were taken regularly for the constipation. The diet consisted of peptonised milk for several days, very cautiously changed in a few days for something more substantial. Antiseptic mouthwashes formed part of the treatment, used both before and after meals. The results are described as highly successful; naturally such a régime is only advisable in exceptionally bad cases.

* *Brit. Med. Jour.*, July 8, 1911.

